# Utilisation of fine needle aspiration cytology and biopsy in Sokoto, Nigeria: A five-year review

**DOI:** 10.4102/ajlm.v8i1.809

**Published:** 2019-05-30

**Authors:** Kabiru Abdullahi, Mohammed Umar, Saddiku M. Sahabi

**Affiliations:** 1 Department of Morbid Anatomy & Forensic Medicine, Faculty of Basic Clinical Sciences, College of Health Sciences, Usmanu Danfodiyo University, Sokoto, Nigeria; 2 Department of Histopathology, Usmanu Danfodiyo University Teaching Hospital, Sokoto, Nigeria

**Keywords:** fine needle, aspirate, cytology

## Abstract

The histopathology department of Usmanu Danfodiyo University Teaching Hospital in Sokoto, Nigeria, conducted a total of 1435 fine needle aspiration biopsies from 1 January 2011 to 31 December 2015, constituting 30% of cytology specimens received. The most common site for the procedure was the breast (655 cases, 45.6%), and 893 cases (63.3%) were neoplastic lesions. Even in resource-poor settings, this underutilised procedure is useful for patient management.

## Introduction

Fine needle aspiration cytology (FNAC) refers to a procedure whereby cells are obtained from a lump, mass or suspicious lesion using a ‘fine bore’ needle (22–27 G) with the aim of microscopic examination of the cells after appropriate staining techniques. The FNAC procedure may be done with or without the use of a syringe. It may be performed guided by palpation or by an ultrasound scan.^1,2,3,4,5^

The first reports of the FNAC procedure date back to the 19th century and since then, there have been tremendous developments in its acceptability and utility as part of the diagnostic armamentarium.^3,4,5^ The procedure is a simple, cheap, reliable, fast and fairly accurate tool, which, in most parts of the developed world, has become routine in medical practice.^2^ However, this may not be the case in resource-constrained settings; for example, it has been underutilised in Nigeria, as reported by Malami and others.^4,6^

There is a need to increase awareness of the procedure, especially with regard to its pivotal role as a cost-effective means of dramatically modifying the course of disease management in patients suspected of having neoplasms. Also, information about its availability and usefulness will assist healthcare policymakers to channel resources towards developing this field of pathology practice in developing countries.^6,7,8^

Virtually every organ or region of the body is accessible to the fine needle for aspiration biopsy. It is minimally invasive, gives the least discomfort (even in children) and does not require elaborate patient preparation, such as anaesthesia. Results can be available within a few hours.^5,6,8,9^

## Methods

### Ethical considerations

Ethical clearance for the study was sought and obtained from the Hospital Research and Ethics Committee of Usman Danfodiyo University Teaching Hospital, Sokoto, Nigeria. The ethical approval number is: UDUTH/HREC/2018/649.

### Setting

The study was conducted in the Department of Histopathology at Usmanu Danfodiyo University Teaching Hospital, Sokoto, a tertiary health institution situated in the north-west region of Nigeria. It provides tertiary healthcare services to (but not exclusively) the Sokoto, Kebbi, Zamfara and Niger states. It also receives referrals from the Republic of Niger, a neighbouring country. Services rendered in the histopathology department of the hospital include histopathology of surgical biopsies and research specimens, cytopathology (including FNAC), frozen section, immunohistochemistry and autopsy.

### Population

Most of the patients presented at the histopathology department for the procedure were on referral after initially being seen by attending physicians in the outpatient units. A few were inpatients, which required the histopathologist to perform the FNAC in the wards. In both cases, the requesting physicians filled out histopathology requisition forms indicating the site for the procedure and other relevant clinical details. The fine needle aspiration was done by the histopathologist, predominantly using a palpation guide.

### Sampling

Universal sampling was employed, in which all cases registered as fine needle aspiration biopsy in the departmental records (reception registers, bench books, request forms, etc.) over the period 1 January 2011 to 31 December 2015 (five years) were consecutively selected specifically with respect to the patients’ age, sex, and site of biopsy. Corresponding microscope slides prepared in quadruplicate (each replicate stained with Papanicolaou stain, Giemsa and hematoxylin or eosin) were retrieved from the departmental archive and re-viewed via light microscopy, using organ specific guidelines. Faded slides were re-stained where possible; missing slides were excluded from the study.

The data generated were entered into a Microsoft Excel 2007 edition (Microsoft Corporation, Redmond Washington, United States) spreadsheet, validated, exported to SPSS version 20 (IBM SPSS Statics for Windows, Version 20.0; IBM Corp, Armonk, New York, United States) for analysis and results were presented as frequency distribution tables.

## Results

Over the five-year period of the study, a total of 1685 FNAC procedures were performed, of which 1435 were included in the study. This represented 30.0% of all cytology specimens seen during the same period. The age range of patients was two weeks to 100 years, with a mean age of 32.7 years.

The procedure was most frequently performed in patients within the age range of 21–30 years and least performed in those less than one year old (Table 1). A male-to-female sex ratio of approximately 1:3 was observed, depicting a female preponderance. This ratio changed to approximately 1:1 when breast FNAC was excluded.

**TABLE 1:** Sex and age distribution of fine needle aspiration biopsy in Sokoto.

Variable	Frequency	Percentage
**Sex**		
Male	391	27.2
Female	1043	72.7
Unspecified	1	0.1
Total	1435	100.0
**Age group**		
< 1year	9	0.6
1–10	124	8.6
11–20	230	16.0
21–30	364	25.4
31–40	288	20.0
41–50	219	15.4
51–60	118	8.2
61–70	52	3.6
71+	17	1.2
Unspecified	14	1.0
Total	1435	100.0

The breasts were the most common site for FNAC, with 655 (45.6%) cases, followed by 378 (26.3%) head and neck cases and 206 (14.4%) lymph node cases. Abdominal viscera were the least accessed organs with 27 (1.9%) cases (Table 2). Non-neoplastic lesions (‘inflammation’ and ‘reactive’) accounted for a total of 250 (17.4%) cases, while neoplastic lesions (‘benign’ and ‘malignant’) accounted for 893 (62.3%) cases. Specifically, there were 740 (51.6%) benign lesions and 153 (10.7%) malignant lesions. There were 218 (15.2%) lesions with suspicious features.

**TABLE 2:** Distribution of fine needle aspiration biopsy by diagnosis, site and malignant cytologic diagnosis in Sokoto.

Variable	Frequency	Percentage
**Diagnosis**		
Acellular/inadequate	74	5.1
Inflammation	212	14.8
Reactive	38	2.6
Suspicious	218	15.2
Benign	740	51.6
Malignant	153	10.7
Total	1435	100.0
**Site/region/organ**		
Head & neck	378	26.3
Ocular	31	2.2
Breast	655	45.6
Lymph node	206	14.4
Soft tissue & osteoarticular	136	9.5
Viscera	27	1.9
Unspecified	2	0.1
Total	1435	100.0
**Site/region/organ (of malignant cytology)**		
Breast	64	41.8
Head & neck	39	25.5
Lymph nodes	16	10.5
Ocular	18	11.8
Soft tissue & osteoarticular	9	5.9
Viscera	7	4.6
Total	153	100.0

## Discussion

A total of 1435 FNAC procedures were performed over the five-year period studied, constituting approximately 30% of all cytology specimens seen in the department. This figure is much higher than that reported from north-central Nigeria where a rate of 97 FNAC per year (over three years) was observed.^9^ However, it is comparable to Faduliye’s study from south-west Nigeria, where a total of 1855 cases (representing FNAC specimens and cytology specimens obtained by other means) were performed over a period of six years (although that study also included cytology specimens obtained by other means).^1^ These studies show that the procedure is still in the early stages of widespread use as compared to figures from centres in developed countries that report up to 1000 cases per year.^5^

We observed that the highest frequencies were seen in the 21–30 years and 31–40 years age ranges. FNAC was also done in individuals at extreme ages (9 [0.6%] who were younger than 1 year and 17 [1.2%] who were older than 71 years) showing that the procedure is applicable irrespective of the patient’s age. These compare favourably with studies from Nigeria and other countries.^1,5,9,10^

The frequency of FNAC among women was higher with 1043 (72.1%) cases as compared to men, and this may reflect the higher frequency of breast FNAC, which accounted for 655 (45.6%) of all FNAC cases. This finding compares with the observations of Mohammed et al. from north-east Nigeria and Vhriterhue from north central Nigeria.^7,8,9^

Only 153 (10.7%) of the cases were malignant, whereas 740 (51.6%) were benign. This is an important feature to note as this outcome underscores the usefulness of the procedure for the immediate triage of patients, which significantly alters the clinical management outline. Other workers have also noticed a similar usefulness of the procedure.^1,8,9,11^

As Vhriterhue and others have noted, a high frequency of ‘grey zone’ diagnosis (e.g. ‘indeterminate’) potentially undermines the usefulness of the FNAC diagnosis. In our centre, we had 15.2% of such ‘grey zone’ (‘suspicious’) cases and this needs to be improved upon to reduce the dilemma and further distress placed on surgeons and patients.^9,11^ To achieve this, it is recommended that the FNAC (especially for barely palpable lesions or lesions located in anatomically difficult sites to access) be performed under ultrasound guide, for example. This will further improve the yield of the aspiration.

### Conclusion

Fine needle aspiration cytology is a useful tool in medical practice. Even in nascent centres like ours, in north-west Nigeria, it has found a place in pathology practice. It can be performed on all patients irrespective of age and sex. It is a cost-effective simple procedure, not usually requiring anaesthesia and can be performed on outpatients as well as inpatients, usually with the pathologist being guided by palpation of the lesion. Its outcome immediately makes a difference in clinical judgment and patient management. It is recommended that there should be capacity building by encouraging the training and retraining of pathologists, and thus encouraging sub-specialisation in this underutilised area of pathology in centres practising in resource-poor regions. In the same vein, resources need to be channelled towards establishing FNAC clinics even at the level of primary healthcare, thereby preventing the frequently reoccurring theme of patients presenting with advanced stage malignant neoplasms. There is a need to correlate cytological and histological findings to further encourage confidence in the procedure.
